# Revisiting the pathogenic mechanism of the *GJB1* 5’ UTR c.-103C > T mutation causing CMTX1

**DOI:** 10.1007/s10048-021-00650-9

**Published:** 2021-06-05

**Authors:** Bianca R. Grosz, John Svaren, Gonzalo Perez-Siles, Garth A. Nicholson, Marina L. Kennerson

**Affiliations:** 1 Northcott Neuroscience Laboratory, ANZAC Research Institute, Concord, NSW Australia; 2 Sydney Medical School, University of Sydney, Camperdown, NSW Australia; 3 Waisman Center, University of Wisconsin-Madison, Madison, WI USA; 4 Department of Comparative Biosciences, University of Wisconsin-Madison, Madison, WI USA; 5 Molecular Medicine Laboratory, Concord Repatriation General Hospital, Concord, NSW Australia

**Keywords:** Charcot-Marie-Tooth, IRES, Neuropathy, Cap-independent translation, CMTX1

## Abstract

The second most common form of Charcot-Marie-Tooth neuropathy (CMT), X-linked CMT type X1 (CMTX1), is caused by coding and non-coding mutations in the gap junction beta 1 (*GJB1*) gene. The non-coding *GJB1* c.-103C > T mutation (NM_000166.5) has been reported to cause CMTX1 in multiple families. This study assessed the internal ribosomal entry site (IRES) activity previously reported for the rat *Gjb1* P2 5’ untranslated region (UTR). Using a bicistronic assay and transfecting RT4 Schwann cells, IRES activity of the human *GJB1* P2 5’ UTR was compared to the *GJB1* P2 5’ UTR containing either the c.-103C > T mutation or the non-pathogenic c.-102G > A variant. No differences in *GJB1* P2 5’ UTR IRES activity were observed between the negative control, the wild-type P2 5’ UTR, the c.-103C > T 5’ UTR or the c.-102G > A 5’ UTR, irrespective of the *GJB1* intron being present (*p* = .429 with intron, and *p* = .865 without). A theoretical c.-131A > G variant was predicted to result in the same RNA secondary structure as the *GJB1* c.-103C > T P2 5’ UTR. However, no significant difference was observed between expression from the wild-type *GJB1* P2 5’ UTR and the *GJB1* c.-131A > G variant (*p* = .688). Deletion of the conserved region surrounding the c.-103C > T mutation (c.-108_-103del) resulted in significantly higher expression than the c.-103C > T mutation alone (*p* = .019), suggesting that the conserved c.-108_-103 region was not essential for translation. The reporter assays in this study do not recapitulate the previously reported *GJB1* IRES activity and suggest an alternate pathogenic mechanism for the c.-103C > T CMTX1 non-coding mutation.

**Supplementary Information:**

The online version contains supplementary material available at 10.1007/s10048-021-00650-9.

## Introduction

Charcot-Marie-Tooth type X1 (CMTX1), the second most common hereditary motor and sensory peripheral neuropathy, is caused by mutations in the gap junction beta 1 (*GJB1*) gene. *GJB1* encodes the transmembrane channel protein connexin 32 (Cx32) and is controlled by two alternative tissue-specific promoters (P1 and P2) that differ in the 5’ untranslated region (UTR) [1]. Non-coding mutations of the neural P2 *GJB1* transcript represent a significant portion of the CMTX1 cohort, with 11.4% of a UK CMTX1 cohort reporting mutations in the neural-specific *GJB1* P2 promoter, 5’ UTR and 3’ UTR [2] (Supplementary Fig. 1). A non-coding pathogenic variant of the neural *GJB1* P2 5’ UTR, c.-103C > T [NM_000166.5, chrX:71,223,249 (hg38)], has been reported in a number of CMTX1 families from multiple ethnic backgrounds [3–11]. Whilst initial studies suggested the c.-103C > T variant completely abolished translation of Cx32 [5], a more recent luciferase-based reporter assay demonstrated the mutation decreased expression by 76.5% when co-transfected with the SOX10 transcription factor [10].

Translation commonly utilises a 5’ cap-dependent mechanism in which ribosomal subunits assemble around the 5′-m_7_G cap end of mature mRNA. In contrast, 5’ cap-independent translation occurs where regions of mRNA, known as an IRES, are able to recruit ribosomal subunits to initiate translation independently of the 5’ cap. It was suggested that the c.-103C > T mutation caused dysfunction of an IRES, as a luciferase reporter assay for the mutation showed that transcription and splicing were not affected, but the translation was abolished [5]. This study suggested that an IRES in the P2 5’ UTR could allow the ribosome to bypass two upstream open reading frames (uORFs) which slow the rate of translation by causing ribosomal stalling [12]. Whilst the vast majority of viral IRES elements are well validated and supported through multiple experimental approaches [13], cellular IRES elements remain contentious and many that have been reported have not been validated using further stringent assays [14–19].

As no consensus structure or sequence exists for cellular or viral IRES elements, they must be determined experimentally. A bicistronic assay allows the direct comparison of 5’ cap-dependent translation and IRES-driven 5’ cap-independent translation by analysing the expression of two reporter genes from a single bicistronic mRNA. Expression of the 5’ cistron relies on 5’ cap-dependent translation of the bicistronic mRNA, whereas expression of the 3’ cistron depends on translation initiation from an intercistronic IRES region. However, the expression of the 3’ cistron may also be due to cryptic promoters or splice sites [14, 18, 20]. Determining that the intended bicistronic RNA is produced by the vector is therefore crucial when asserting IRES function [21].

Secondary structural features of the 5’ UTR are not only crucial for the regulation of translation through IRES mechanisms, but also through aiding recognition of the correct translation start site and modulating the rate of translation [22]. Previous studies have demonstrated that an increase in the G-C content of RNA stem-loops close to the 5’ cap results in a direct decrease in translation efficiency without affecting RNA abundance [23]. This was similar to the previously reported findings for the c.-103C > T mutation [5]. Given that ribosomal scanning following initiation at the 5’ cap is essential for translation initiation [24, 25], it is possible that the previously reported change in the secondary structure induced by the c.-103C > T variant^7^ suggests a possible pathogenic mechanism.

The initial *GJB1* P2 5’ UTR experiments supporting an IRES dysfunction hypothesis were conducted using the rat *Gjb1* P2 5’ UTR [5], which has since been shown to differ in both sequence and secondary structure [7] when compared to human *GJB1* P2 5’ UTR (Supplementary Fig. 2). Additionally, the bicistronic transcript was not confirmed by RNA analysis. The effectiveness of an IRES varies amongst cell types and this is likely due to the need for cell-specific IRES trans-acting factors (ITAFs) to assist in the recruitment of the ribosome [26]. The *GJB1* IRES activity was previously demonstrated by performing bicistronic assays in HeLa cells, mouse fibroblasts (NIH-3T3) and neuroblastoma cell lines (Neuro2a) [5]. As Cx32 is expressed in Schwann cells in the peripheral nervous system, we used a Schwann cell model to reflect an appropriate tissue for CMTX1 that would likely contain the appropriate cell-specific ITAFs.

We have performed a bicistronic assay in RT4 rat Schwann cells and HeLa cells using constructs containing the human wild-type *GJB1* P2 5’ UTR, the pathogenic c.-103C > T mutation and the adjacent non-pathogenic c.-102G > A variant [27]. Given that both sequence and structural motifs are theorised to be crucial for IRES function, additional luciferase reporter assays were developed to assess these aspects of the *GJB1* P2 5’ UTR. An assay was developed to assess the effect of *GJB1* P2 5’ UTR structure motifs on translation, as well as a deletion of the conserved sequence surrounding c.-103C > T to assess the functional effects of this sequence. Collectively, the results of these reporter assays do not support the role of IRES dysfunction as the pathogenic mechanism for the *GJB1* c.-103C > T mutation.

## Results

**Fig. 1:**
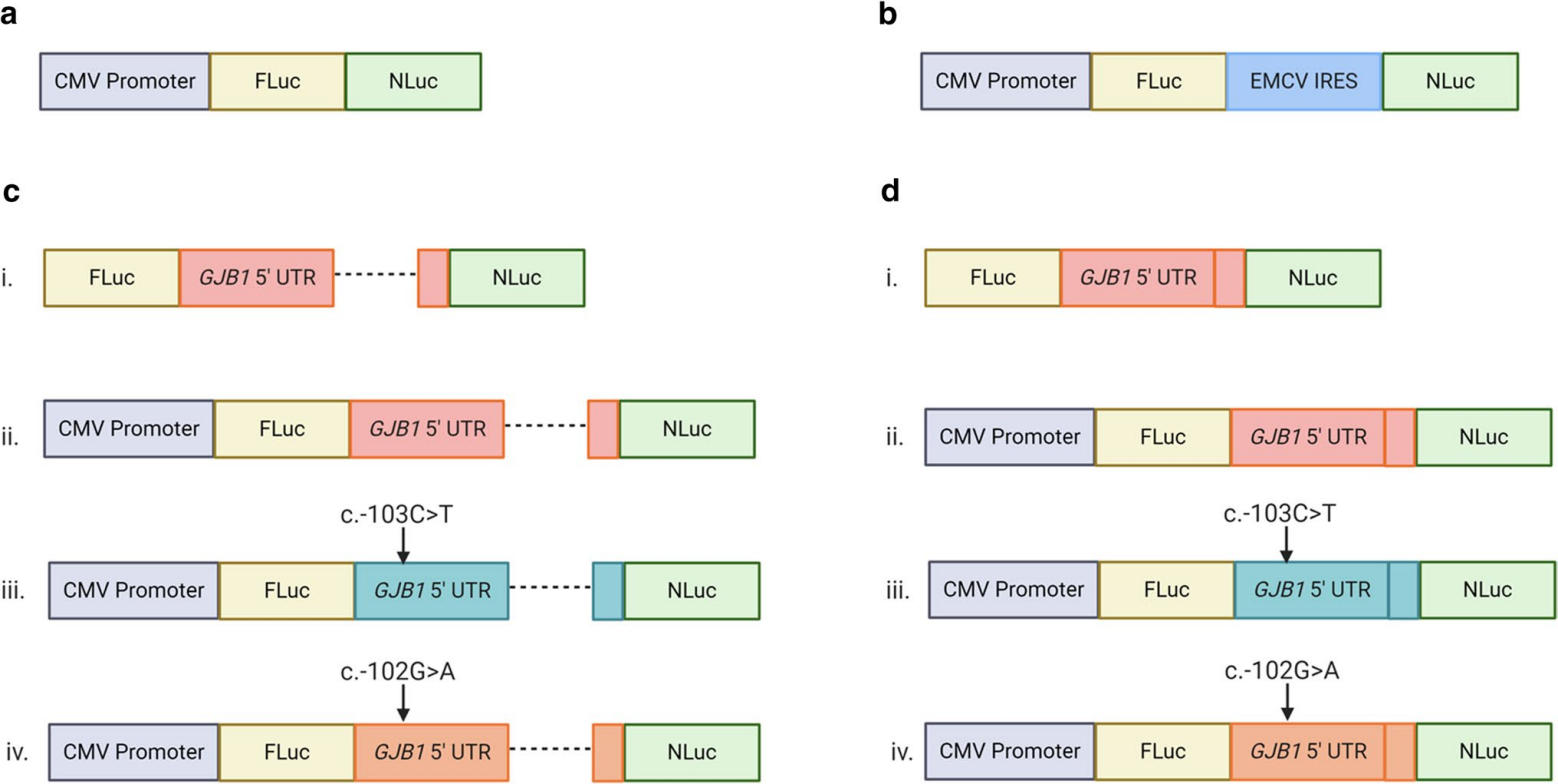
*GJB1* bicistronic assay design for the assessment of IRES activity. **a** A bicistronic vector with no intercistronic IRES serves as a negative control. **b** A vector containing the well-characterised viral EMCV IRES inserted between FLuc and NLuc acts as a positive control for IRES activity. **c** The wild-type *GJB1* P2 5’ UTR (ii) is inserted between the cytomegalovirus (CMV) promoter-controlled firefly luciferase (FLuc) reporter gene, and the NanoLuc luciferase (NLuc) reporter gene, as well separate *GJB1* P2 5’ UTR vectors harbouring the pathogenic c.-103C > T *GJB1* (iii), and non-pathogenic c.-102G > A *GJB1* (iv). Created with BioRender.com. **d** A series of experimental bicistronic assays were also created with a deletion of the *GJB1* intron

The pathogenic c.-103C > T variant occurs in the 5’ UTR of the P2 neural-specific transcript of *GJB1* and causes CMTX1. A bicistronic assay was performed to reassess the previously reported IRES activity of the rat *Gjb1* P2 5’ UTR and the effect of the pathogenic c.-103C > T and non-pathogenic c.-102G > A variants. Bicistronic vectors were designed in which translation of the first reporter gene (firefly luciferase; FLuc) was 5’ cap-dependent, and translation of the second reporter gene (NanoLuc luciferase; NLuc) required the human *GJB1* P2 5’ UTR to function as an IRES and initiate translation through a 5’ cap-independent mechanism (Fig. 1). The activity of FLuc acts as a control for cell viability and transfection efficiency and NLuc activity suggests IRES function of the intercistronic sequence. The known IRES from the encephalomyocarditis virus (EMCV) was used as an IRES-positive control (Fig. 1b), as this demonstrated similar IRES expression to the *Gjb1* P2 5’ UTR previously reported [5]. Bicistronic vectors containing the full P2 5’ UTR (Fig. 1c) and the P2 5’ UTR with the 356 bp *GJB1* intron deletion (Fig. 1d) were used for separate transient transfections into the RT4 Schwann cell line. The bicistronic vector containing the *GJB1* P2 5’ UTR intron deletion (Fig. 1d) was transiently transfected into the HeLa cell line to provide a direct comparison to the previously published findings [5].

**Fig. 2:**
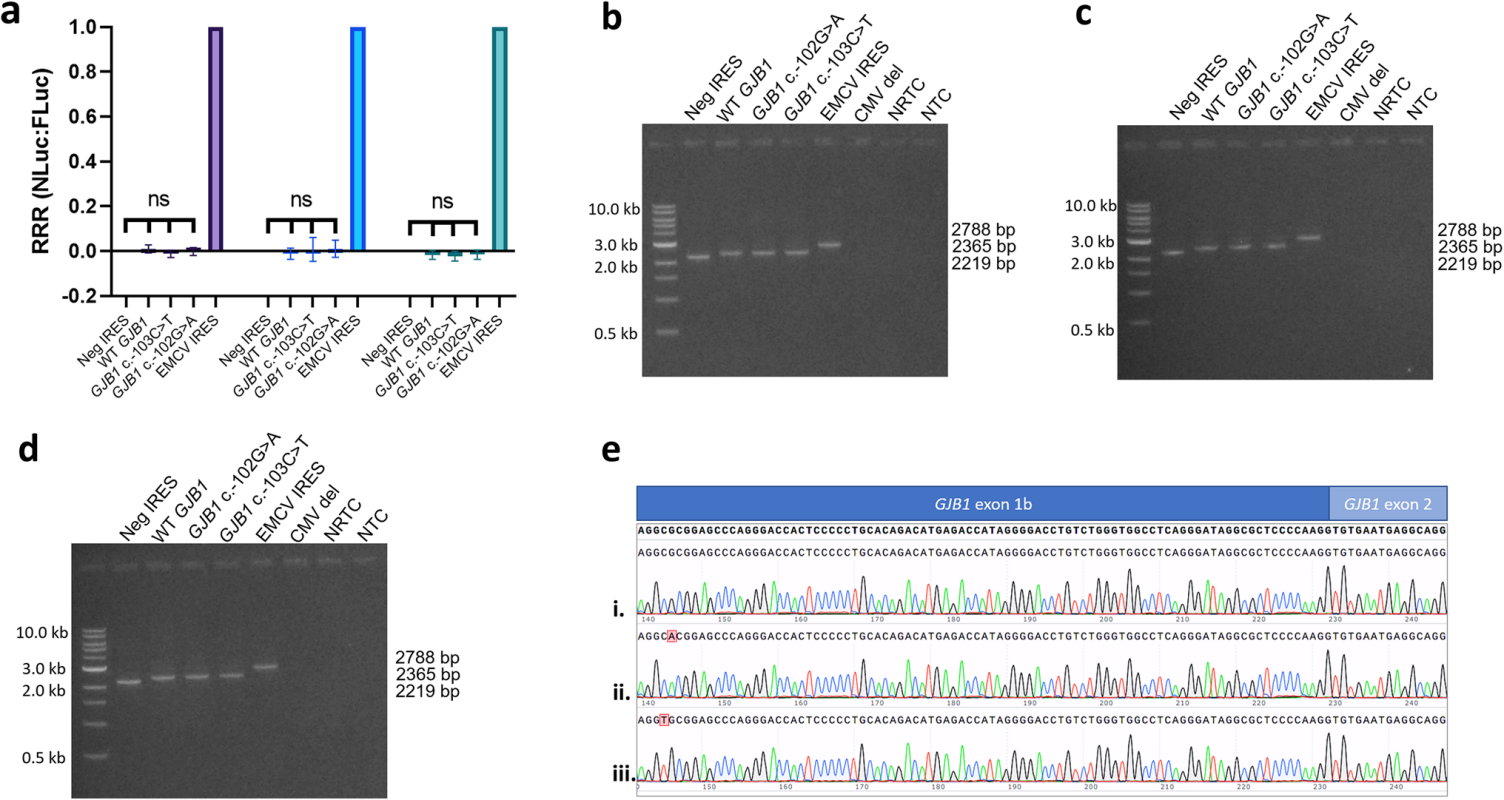
The bicistronic assay to assess IRES activity of the *GJB1* P2 5’ UTR shows no evidence of increased translation of NLuc when compared to the negative IRES construct. **a** There was no statistically significant difference in IRES activity between the negative control, the wild-type *GJB1* P2 5’ UTR, the *GJB1* c.-103C > T 5’ UTR, *GJB1* c.-102G > A 5’ UTR in RT4 Schwann cells when vectors contained the *GJB1* intron (purple) and without the *GJB1* intron (blue). There remained no significant difference when the intronless bicistronic assay was conducted in HeLa cells (green). The relative response ratio (RRR) for each vector is shown on the y-axis, with the error bars indicating standard deviation. Statistical significance was assessed using a one-way ANOVA. Bicistronic mRNA from each vector was present for RT4 cells with *GJB1* bicistronic vectors containing the intron (**b**), RT4 cells transfected with *GJB1* bicistronic vectors with the intron deleted (**c**) and HeLa cells transfected with *GJB1* bicistronic vectors with the intron deleted (**d**). A positive band is visible for the negative IRES vector (2219 bp), the three experimental *GJB1* P2 5’ UTR vectors (2365 bp) and the positive EMCV IRES vector (2788 bp). No band was evident in the vector with the CMV promoter deletions (CMV del). NTC indicates a negative PCR reaction control (no template control). NRTC indicates a negative cDNA conversion control (no reverse transcriptase control). Size ladder (lane 1) NEB 1-kb DNA ladder. Created with BioRender.com. **e** Sanger sequencing confirmed correct splicing of the *GJB1* P2 5’ UTR for wild type (i), c.-102G>A (ii), and c.-103C>T (iii) bicistronic mRNA

The bicistronic assay containing the 356 bp intron (Fig. 2a) showed no significant differences in translation initiation when compared to all permutations of the *GJB1* P2 5’ UTR bicistronic vectors (wild type, c.-103C > T, c.-102G > A) and negative control (F(3,8) = 1.03, *p* = 0.429). Similarly, the bicistronic assay results without the 356 bp intron in RT4 cells (F(3,8) = 0.242, *p* = 0.865) and HeLa cells (F(3,8) = 0.845, *p* = 0.506) showed no significant difference when compared to the negative control (Fig. 2a). These results suggest the wild-type *GJB1* P2 5’ UTR does not initiate 5’ cap-independent translation.

To demonstrate transcription of a complete bicistronic mRNA for the designed constructs, cDNA templates from cells transfected with each of the bicistronic constructs were analysed. Primers amplifying the transcribed full-length bicistronic mRNA showed the expected amplicon sizes for the negative IRES control (2219 bp; Fig. 2b–d), the experimental *GJB1* P2 5’ UTR constructs (2365 bp; Fig. 2b–d) and the positive EMCV IRES control (2788 bp; Fig. 2b–d). Sanger sequencing further confirmed the correct sequences for the different amplicons transcribed from each bicistronic vector (Fig. 2e). No amplicon was observed for the cDNA synthesis controls with no reverse transcriptase (NRTC), which confirms that the amplicons observed did not result from plasmid contamination.

**Fig. 3:**
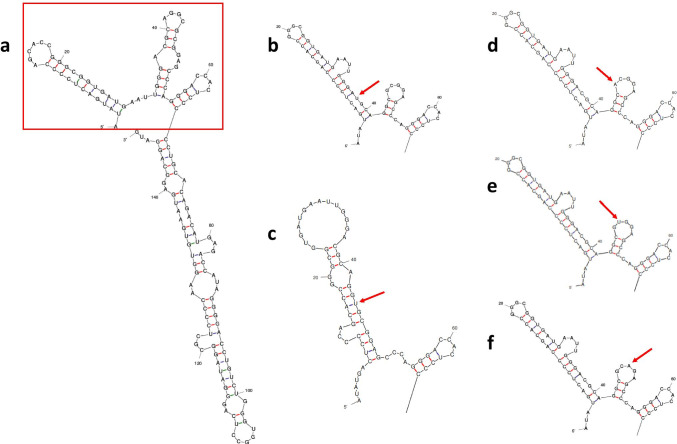
Predicted secondary structures with minimum free energy for the P2 5’ UTR of *GJB1* spliced mRNA using mFold (version 2.4)[28] reveals that the pathogenic c.-103C > T mutation causes a conformational change near the 5’ cap which differs to both the wild-type *GJB1* P2 5’ UTR and surrounding non-pathogenic variants. **a** Wild type. **b** *GJB1* c.-109C > T. **c** *GJB1* c.-103C > T. **d** *GJB1*c.-102G > A. **e** *GJB1*c.-101C > T. **f** *GJB1* c.-100G > A. The most thermodynamically stable secondary structure is shown for each sequence as predicted using default parameters. The region shown for each variant is *GJB1* c.-146 to c.-80 (boxed in red on the wild-type structure), as the predicted secondary structure for each variant is the same as the wild type from c.-80 to the start codon. The substituted base is indicated by an arrow

Given the presence of non-pathogenic sequence variants surrounding the pathogenic c.-103C > T mutation (Table 1), it was theorised that secondary RNA structures may hinder the recruitment of translation machinery. We used mFold (version 2.4) [28] to predict secondary structural changes to the *GJB1* P2 5’ UTR transcript using six sequence changes (Fig. 3) wild-type *GJB1* P2 5’ UTR (Fig. 3a), *GJB1* c.-109C > T (Fig. 3b), *GJB1* c.-103C > T (Fig. 3c), *GJB1* c.-102G > A (Fig. 3d), *GJB1* c.-101C > T (Fig. 3e) and *GJB1* c.-100G > A (Fig. 3f). The introduction of the non-pathogenic variants was predicted to lengthen a 5’ hairpin and shortened the subsequent hairpin when compared to the wild-type secondary structure. However, the introduction of the c.-103C > T mutation generated a hairpin close to the 5’ cap for which 10/13 base pairs in the stem were stable pairings between guanine and cytosine (indicated by red lines). In contrast, for the wild-type structure, 6/11 stem base pairs were G-C, and for all the other variants assessed 8/15 stem pairs were G-C. This suggested the unique RNA secondary structure caused by the *GJB1* c.-103C > T mutation may reflect a possible mechanism for pathogenicity.

**Table 1:** Reported SNPs in the *GJB1* P2 5’ UTR surrounding the pathogenic *GJB1* c.-103C > T variant

SNP ID	Coding change (NM_000166.5)	Clinical significance (Clinvar)	TOPMed [44]	GnomAD [45]
rs746618959	c.-109C > T	Not reported	73/125568	5/21580
rs753207004	c.-102G > A	Not reported	3/125568	1/21640
rs961829342	c.-101C > T	Likely benign (RCV000426678.1)	15/125568	2/21616
rs961626121	c.-100G > A	Not reported	6/125568	2/21621

**Fig. 4:**
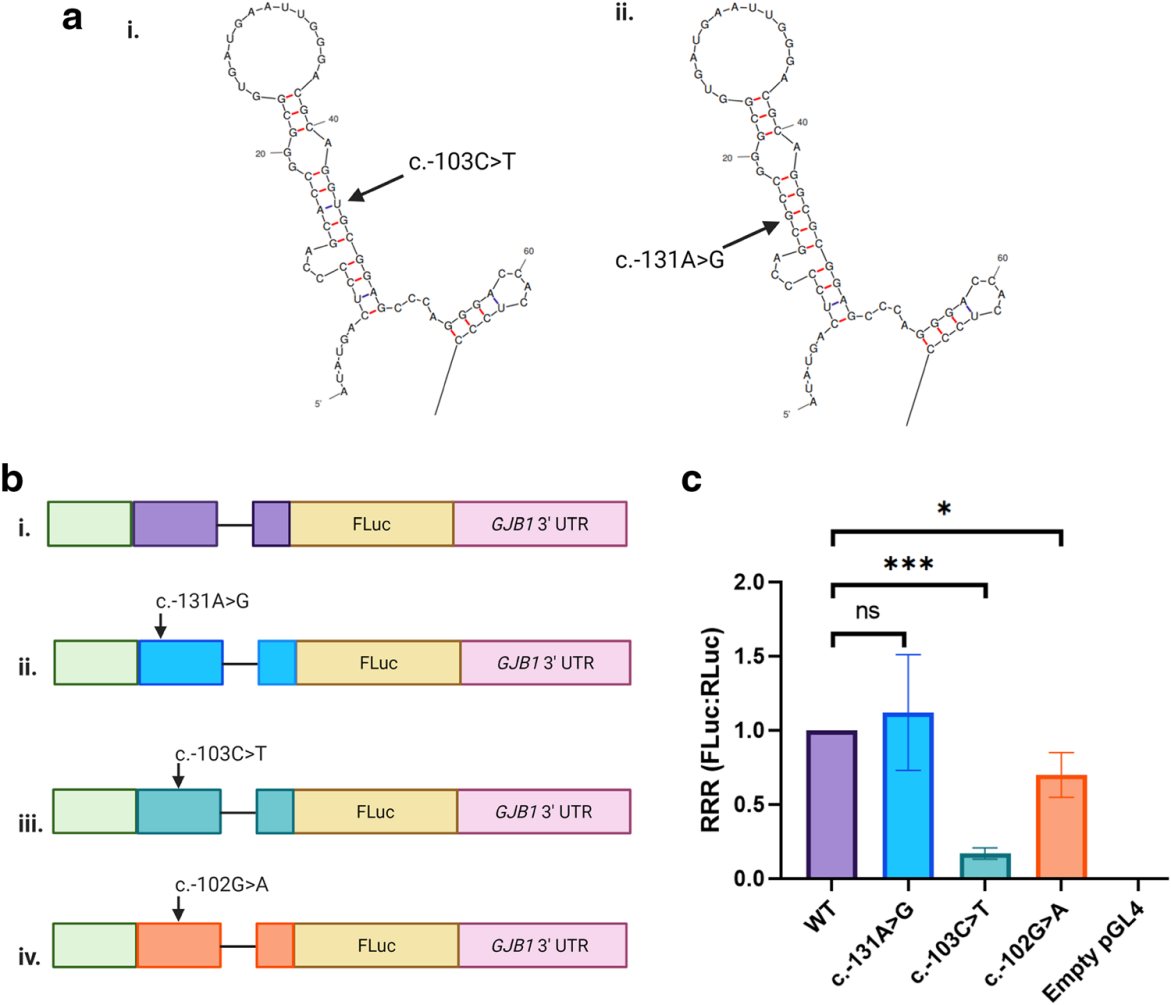
*GJB1* c.-131A > G results in the same *GJB1* 5’ UTR RNA secondary structure as *GJB1* c.-103C > T but does not affect expression. **a** The *GJB1* c.-131A > G variant was predicted to generate the same RNA secondary structure of the *GJB1* P2 5’ UTR as the *GJB1* c.-103C > T mutation. **b** pGL4-based constructs were generated where expression of FLuc was controlled by the (i) wild-type *GJB1* P2 promoter and 5’ UTR (purple), as well as the *GJB1* 3’ UTR. The (ii) *GJB1* c.-131A > G (blue), (iii) *GJB1* c.-103C > T (green) and (iv) *GJB1* c.-102G > A (orange) substitutions were introduced into the *GJB1* 5’ UTR. **c** There was no difference between the FLuc expression from the *GJB1* c.-131A > G substitution and wild-type *GJB1* 5’ UTR (p = .688). There was a significant difference between the FLuc expression from the wild-type *GJB1* P2 5’ UTR and both *GJB1* c.-102G > A (p < 0.048) and *GJB1* c.-103C > T (p < 0.00001). The relative response ratio (RRR) for each vector is shown on the y-axis, with the error bars indicating standard deviation. Statistical significance was assessed using a two-tailed t-test. Created with BioRender.com

To investigate if changes to the RNA secondary structure of the *GJB1* P2 5’ UTR caused by c.-103C > T were pathogenic, a nucleotide substitution was introduced that predicted the same *GJB1* 5’ UTR RNA secondary structure as the c.-103C > T mutation. The c.-131A nucleotide forms the stem base pair opposite to *GJB1* c.-103C > T, and therefore, the substitution *GJB1* c.-131A > G was predicted by mFold to have the same RNA secondary structure as the c.-103CT > *GJB1* P2 5’ UTR (Fig. 4a and b). pGL4-based luciferase constructs were cloned in which the FLuc gene was flanked by the *GJB1* P2 promoter and 5’ UTR and the *GJB1* 3’ UTR (Fig. 4b). The different *GJB1* substitutions c.-131A > G (b.ii.), c.-103C > T (b.iii.) and non-pathogenic c.-102G > A (b.iv.) were introduced into the 5’ UTR. Using pRL-TK as a transfection control, the constructs were transfected separately into the RT4 cell line and FLuc and RLuc expression was measured. There was a significant decrease in expression due to the *GJB1* c.-103C > T mutation (*M* = 0.16, *SD* = 0.04) when compared to the wild-type *GJB1* P2 5’ UTR (*p* < 0.00001) (Fig. 4). However, there was no difference between the FLuc expression from the *GJB1* c.-131A > G substitution (*M* = 1.12, *SD* = 0.48) and wild-type *GJB1* 5’ UTR (*p* = 0.688). There was a significant difference between the FLuc expression from *GJB1* c.-102G > A (*M* = 0.70, *SD* = 0.18) and the wild-type *GJB1* P2 5’ UTR (p < 0.048), although this variant had been reported in individuals without CMTX1.

**Fig. 5:**
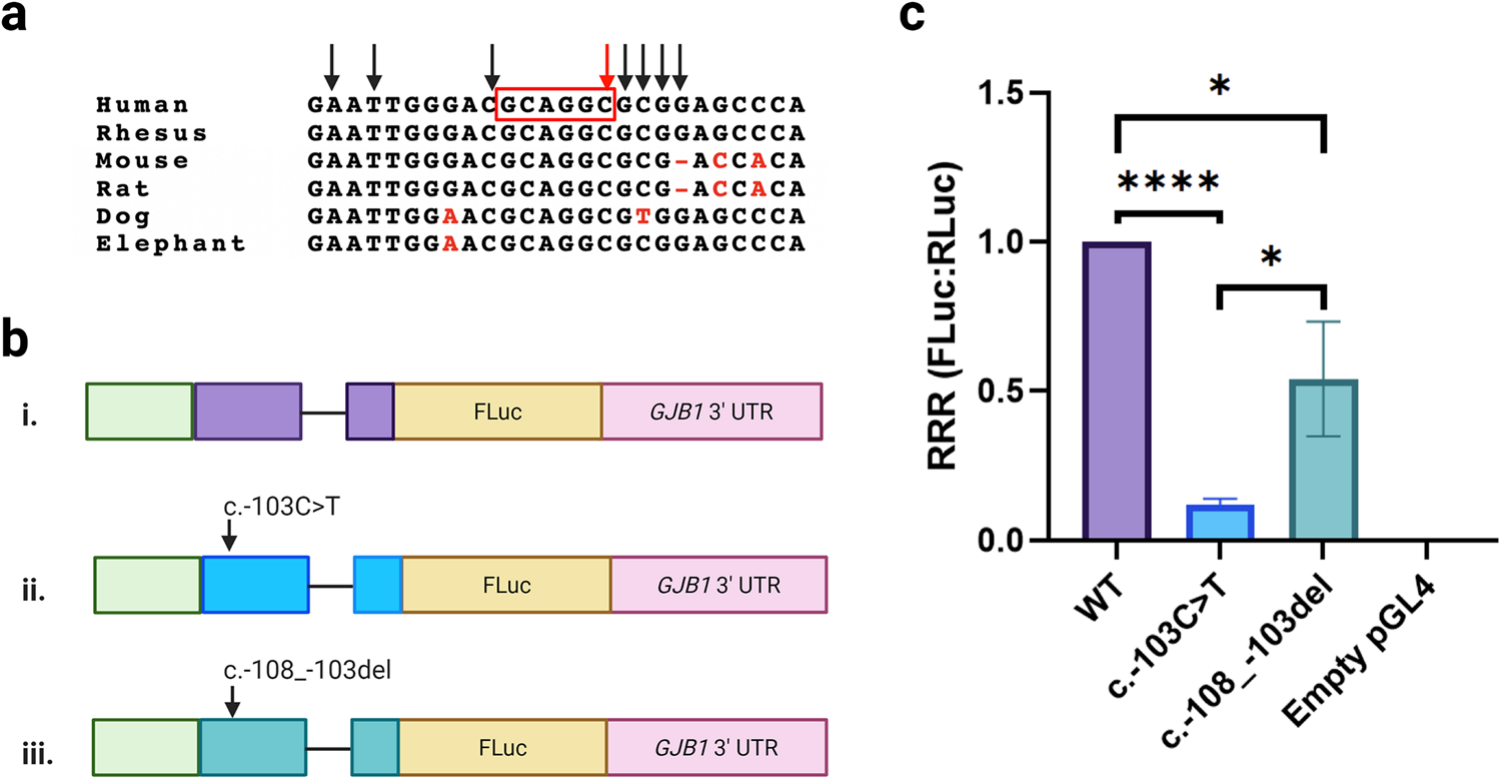
The *GJB1* c.-103C > T mutation is within an evolutionarily conserved region; however, deletion of this region did not decrease expression to *GJB1* c.-103C > T levels. **a** *GJB1* c.-103C > T (red arrow) is the last base in a 6 bp region (c.-108_-103) that is evolutionarily conserved across vertebrate species. Bases which are not conserved are highlighted in red and bases in which single-nucleotide polymorphisms have been reported in population databases are indicated by black arrows. **b** pGL4-based constructs were generated where expression of FLuc was controlled by the (i) wild-type *GJB1* P2 promoter and 5’ UTR (purple), as well as the *GJB1* 3’ UTR. The (ii) *GJB1* c.-103C > T (blue) substitution was previously introduced into the 5’ UTR. (iii) The *GJB1* c.-108_-103del (green) deletion was introduced into the *GJB1* 5’ UTR to assess the functional effect of this region. **c** The *GJB1* c.-108_-103del construct and *GJB1* c.-103C > T both resulted significant decrease in expression when compared to the wild-type *GJB1* construct (p = .014 and p < .00001, respectively). However, the decrease in expression due to *GJB1* c.-108_-103del was significantly different from the decrease in expression due to the c.-103C > T mutation (p = .019). The relative response ratio (RRR) for each vector is shown on the y-axis, with the error bars indicating standard deviation. Statistical significance was assessed using a two-tailed t-test. Created with BioRender.com

The *GJB1* c.-103C > T mutation occurs in an evolutionarily conserved region and is flanked by two non-pathogenic SNPs, c.-109C > T (rs746618959) and c.-102G > A (rs753207004) (Fig. 5a). To determine if this region represents a regulatory region that is abolished by the *GJB1* c.-103C > T mutation, an additional pGL4-based luciferase deletion construct *GJB1* c.-108_-103del was cloned (Fig. 5b). The *GJB1* c.-108_-103del construct resulted in a 46% decrease (*M* = 0.54, *SD* = 0.19) in expression when compared to the wild-type *GJB1* construct (p = 0.014) (Fig. 5c). However, the *GJB1* c.-103C > T mutation resulted in an 88% decrease (*M* = 0.12, *SD* = 0.02) in expression when compared to the wild-type *GJB1* construct (p < 0.00001). Although *GJB1* c.-108_-103del showed a significant decrease in expression when compared to wild type, it remained significantly different from the *GJB1* c.-103C > T mutation (p = 0.019). This result suggests that abolishing the conserved region (c.-108 to c.-103) alone is not likely to explain the full pathogenic mechanism of the *GJB1* c.-103C > T mutation.

## Discussion

Previous studies examining the *GJB1* c.-103C > T mutation proposed that it abolished an IRES in the *GJB1* P2 5’ UTR. As more IRES elements have been reported, however, the validity of many cellular IRES elements has been questioned [14–19]. The results of the bicistronic assay in our study do not support the previously proposed IRES dysfunction as a pathogenic mechanism for the *GJB1* c.-103C > T mutation causing CMTX1. Given the contentious nature of findings in the IRES literature, it was essential for this study to repeat the bicistronic assay using the human transcript of *GJB1*, using the appropriate controls, and providing evidence that a full-length bicistronic mRNA was being produced in the assays. To develop effective therapeutic approaches for CMTX1 patients harbouring the *GJB1* c.-103C > T mutation, it is imperative that the underlying mechanism for the mutation could be validated and experimentally reproduced. Our results have clearly shown that abolishing an IRES element is highly unlikely to be the pathogenic mechanism.

The previously published bicistronic vectors which supported an IRES element in the *GJB1* P2 5’ UTR contained an initial stable stem-loop structure preceding the cap-dependent RLuc, and a second stable stem-loop structure preceding the *GJB1* P2 5’ UTR and cap-independent FLuc. Although not experimentally confirmed, it was suggested that the initial stem-loop would reduce high levels of cap-dependent translation of RLuc, and the second stem-loop would reduce ribosomal readthrough and therefore background FLuc [5]. Comparatively, our bicistronic assay calculated the ratio of cap-dependent FLuc and cap-independent NLuc and no stable stem-loop structures were used. As the high-intensity luminescence produced by NLuc is ~ 150-fold greater than both FLuc and RLuc [29], an initial stable stem-loop to reduce cap-dependent FLuc expression was unnecessary. As it has been shown that IRES elements are canonically complex structures containing a series of RNA stem-loops [30, 31], we further hypothesised that a stable stem-loop preceding the *GJB1* P2 5’ UTR could promote false IRES activity. This was previously reported for the *pim-1* 5’ UTR, which showed ‘IRES activity’ when assessed in a bicistronic vector with a series of stem-loops preceding the *pim-1* 5’ UTR [32]. However, when the *pim-1* 5’ UTR was stringently reassessed without these stem-loops both in vitro and in vivo, the IRES activity was no longer observed [18]. Furthermore, our modelling of the *GJB1* P2 5’ UTR suggested that RNA secondary structure may be crucial for its IRES function. Therefore, as suggested in the critical review of IRES validation [33], we instead utilised a series of intercistronic stop codons and ensured NLuc was out of frame with FLuc to mitigate continued ribosomal scanning of the bicistronic RNA following FLuc termination.

In this study, EMCV IRES was used as a positive control. The EMCV IRES has previously been shown to stably mediate 5’ cap-independent translation in both HeLa and NIH3T3 cells [14] and was previously reported to have similar levels of IRES activity as the *GJB1* P2 5’ UTR IRES [5]. The bicistronic assay designed for this study used the human *GJB1* P2 5’ UTR in RT4 rat Schwann cells with the hypothesis that the RT4 line would contain Schwann cell-specific ITAFs and best replicate the tissue affected in CMTX1 patients. Our bicistronic assays were also conducted in HeLa cells, as this was a cell line in which the *GJB1* P2 5’ UTR bicistronic assay was previously conducted [5] and we were again unable to replicate the previous IRES finding.

To confirm a functional IRES, it is crucial to prove that only the intended bicistronic RNA is produced by the bicistronic vector. The initial characterisation of the *GJB1* P2 5’ UTR IRES did not include RNA analysis following transfection with the pRLuc-FLuc bicistronic vectors. It is therefore possible that cryptic promoter activity or unintended splicing of the bicistronic mRNA could result in the unintended translation of the 3’ cistron. Previous studies have shown that the use of a pRLuc-FLuc constructs can lead to the identification of false positive IRES elements, due to spurious splicing events caused by the presence of a chimeric intron in RLuc and strong splice donor sites in the pRLuc-FLuc vector [16, 34]. The 5’ UTR of the X-linked inhibitor of apoptosis (XIAP) is an example showing purported IRES activity in a pRLuc-FLuc vector; however, cryptic promoter activity and splicing were demonstrated to mediate this apparent IRES activity [14, 17, 34, 35]. The removal of 5’ splice donor sites upstream of a putative IRES in a pGluc-GFP bicistronic vector removed the apparent IRES activity of four of six reported eukaryotic IRES due to the presence of a 3’ splice acceptor site within the putative IRES sequence [14]. It was previously determined that mutating a polypyrimidine tract of the *GJB1* P2 5’ UTR could increase its apparent IRES activity; however, polypyrimidine tracts are also known to be part of the consensus sequence that identifies a 3’ splice acceptor site [36].

Studies have also suggested that the 5’ UTRs of both Cx26 and Cx43 contain an IRES, and these experiments were conducted using the same bicistronic vector system as for Cx32 (pRLuc-FLuc) [37, 38]. However, the possibility of a cryptic promoter was only excluded for Cx26 [37]. Recent attempts to recapitulate the previously reported IRES activity of three isoforms of the Cx43 5’ UTR were unsuccessful [39]. The Cx43 transcript has been reported to contain an internal IRES leading to translation of a smaller 20 kDa Cx43 isoform; however, the subsequent analysis determined that upstream scanning or 5’ cap-dependent translation by the ribosome is necessary to initiate this internal translation [15]. It is possible that the 5’ UTR of *GJB1* may act in the same way, where elements of the 5’ UTR are required to regulate translation following the canonical recognition of the 5’ cap by the ribosome and therefore is not truly ‘cap independent’.

There are several lines of evidence which do not support the role of IRES dysfunction in the pathogenicity of the *GJB1* c.-103C > T mutation in CMTX1. Initially, it was suggested that the *GJB1* c.-103C > T mutation was immediately upstream of a GNRA tetraloop motif sequence (N refers to any nucleotide, R refers to G or A) [5], which is a motif that has been shown to regulate the activity of viral IRES elements [40–42]. However, subsequent iterations of the human reference genome have revealed the insertion of an additional base in this region, and therefore, this motif is no longer present. Whilst deletion of the conserved region upstream of *GJB1* c.-103C > T (c.-108_-103del) did decrease expression when compared to the wild-type *GJB1* P2 5’ UTR, the decrease was significantly different from the c.-103C > T mutation. Additionally, variants in the three bases immediately downstream of the *GJB1* c.-103C > T mutation (*GJB1* c.-102G > A, *GJB1* c.-101C > T, *GJB1* c.-100G > A), as well as six bases upstream (*GJB1* c.-109C > T), represent variants present in databases at frequencies that do not support pathogenicity (Table 1). Collectively, these results do not suggest that recognition of this region is necessary for translation, and therefore, it is unlikely that this region is an IRES required for Cx32 translation. Instead, these results suggest that the *GJB1* c.-103C > T mutation is possibly pathogenic due to the creation of a negative sequence motif, such as a repressor binding site, rather than the disruption of a functionally important element.

Overall, the bicistronic assays designed in this study revealed no evidence of an IRES in the 5’ UTR of *GJB1*, regardless of the presence of the *GJB1* intron or the cell line used. Similarly, the bicistronic vectors containing either the pathogenic (c.-103C > T) or non-pathogenic (c.-102G > A) variant showed no IRES activity. Further luciferase reporter assays did not support aberrant RNA secondary structure or the disruption of a functional regulatory element in the *GJB1* P2 5’ UTR as pathogenic mechanisms for the c.-103C > T mutation. Collectively, these results do not support the role of IRES dysfunction as the pathogenic mechanism for the *GJB1* c.-103C > T mutation and alternative explanations warrant consideration. Further investigation to fully define a pathogenic mechanism will enable suitable strategies for developing treatment therapies for CMTX1.

## Materials and methods

### Standard protocol approvals, registrations and patient consents

Individuals participating in this study were enrolled through the Neurogenetics Clinic Concord Hospital, Sydney. Genomic DNA was isolated from peripheral blood. These procedures were performed with informed consent according to protocols approved by the Sydney Local Health District, Human Ethics Committee, Concord Hospital, Australia (HREC/17/CRGH/8).

### Generation of bicistronic luciferase vectors

The existing reporter vector CMV-Xbp1-FLuc2a-NLuc2a-Puro [43] was modified through multiple steps using the Q5® Site-Directed Mutagenesis (SDM) Kit (New England Biolabs, Beverly, MA, USA) to generate the bicistronic vectors in Fig. 1. Transformations were performed with One Shot TOP10 chemically competent *E. coli* (Invitrogen, Carlsbad, CA 92,008 USA) and plasmid DNA was subsequently purified using the Isolate II Plasmid Mini Kit (Bioline). All plasmids underwent Sanger sequencing to verify the introduced mutations and variants using the BigDye Terminator Cycle Sequencing protocols at Garvan Molecular Genetics (Garvan Institute of Medical Research, Australia).

To generate the bicistronic vector pCMV-FLuc-NLuc (Fig. 1b), deletion of Xbp1 from CMV-Xbp1-FLuc2a-NLuc2a-Puro was performed using Xbp1del_F and Xbp1del_R primers that flanked the Xbp1 region (Table 2). Insertion of stop codons downstream of NLuc and deletion of the 2aNeo region was then performed using primers that flanked the 2aNeo region with stop codons added to the 5’ end of the forward primer, 2aNeodel_NLucStop_F and 2aNeodel_NLucStop_R (Table 2). A series of stop codons downstream of FLuc and unique HindIII and AflII restriction sites were inserted in the intercistronic region between FLuc and NLuc through the addition of half of the insertion sequence to the 5’ end of each primer, FLucStop_HindIIIAflII_F and FLucStop_HindIIIAflII_R (Table 2). The stop codons ensured that the ribosome would terminate translation following translation of FLuc, and additional bases ensured that FLuc and NLuc were out of frame to mitigate ribosomal readthrough leading to expression of NLuc [33]. The intercistronic 2a region was then deleted using 2adel_F and 2adel_R (Table 2). This vector served as the negative control (Fig. 1a).

**Table 2:** Primer names and sequences used for the cloning of luciferase reporter vectors

Primer name	Sequence (5’–3’)
Xbp1del_F	TCGCCCATGGAAGATGCC
Xbp1del_R	GCTAGCCAGCTTGGGTCT
2aNeodel_NLucStop_F	taataaGATCGACCGATGCCCTTG
2aNeodel_NLucStop_R	AGCCAGAATGCGTTCGCA
FLucStop_HindIIIAflII_F	gcggcggaagcttGGAGAGGGCAGAGGAAGT
FLucStop_HindIIIAflII_R	ttaagttattattaAGAATTCACGGCGATCTTG
2adel_F	GCTCGAGTTTTTGGCATCTTC
2adel_R	AAGCTTCCGCCGCTTAAG
EMCVIRES_AflII_F	AAAAAAAAGCTTATATGACTCCCCAGCACCGG
EMCVIRES_XhoI_R	AAAAAACTCGAGCCTGCCTCATTCACACCTGCAA
GJB1P25UTR_HindIII_F	AAAAAAAAGCTTATATGACTCCCCAGCACCGG
GJB1P25UTR_XhoI_R	AAAAAACTCGAGCCTGCCTCATTCACACCTGCAA
GJB1_WTSDM_F	GGGACGCAGGcGCGGAGCCCA
GJB1_WTSDM_R	AATTCATCACCGCCCGGTGCTGG
GJB1_102GASDM_F	GGACGCAGGCaCGGAGCCCAG
GJB1_102GASDM_R	CAATTCATCACCGCCCGGTGC
GJB1_Introndel_F	GTGTGAATGAGGCAGGATGATATG
GJB1_Introndel_R	CTTGGGGAGCGCCTATCC
CMVPromdel_F	ATCTGGCTAGCTCGCCCATG
CMVPromdel_R	CGATTCACACAAAAAACCAACAC
GJB1P2Prom5UTR_HindIII_F	AAAAAACTCGAGATCCACCTGCCTGTGTTTTATCTC
GJB1P2Prom5UTR_XhoI_R	AAAAAAAAGCTTCCTGCCTCATTCACACCTGCAA
GJB13UTR_F	AAAAAATCTAGATGCCACATACCAGGCAAC
GJB13UTR_R	AAAAAAGGCCGGCCTTCAGAGGGAGTTGTCATTTTTAATC
HindIII_ATGdel_F	ATGGAAGATGCCAAAAAC
HindIII_ATGdel_R	CCTGCCTCATTCACACCT
GJB1_103CTSDM_F	GGGACGCAGGtGCGGAGCCCA
GJB1_103CTSDM_R	AATTCATCACCGCCCGGTGCTGG
GJB1_131AGSDM_F	ACTCCCCAGCgCCGGGCGGTG
GJB1_131AGSDM_R	CATATGCTGCTTTATACCCAGTGTCTG
GJB1_108103delSDM_F	GCGGAGCCCAGGGACCAC
GJB1_108103delSDM_R	GTCCCAATTCATCACCGCCCG

Mutagenesis sequences are indicated in lower case

A positive IRES control was generated by amplifying the encephalomyocarditis (EMCV) IRES region from pLPCX-Cx43-IRES-GFP [15] using EMCVIRES_AflII_F and EMCVIRES_XhoI_R (Table 2). pLPCX-Cx43-IRES-GFP was a gift from Trond Aasen (Addgene plasmid # 65,433; http://n2t.net/addgene:65433; RRID:Addgene (65,433). The amplicon was cloned into the bicistronic pCMV-FLuc-NLuc vector in the intercistronic region between FLuc and NLuc using the AflII/XhoI sites to generate pCMV-FLuc-EMCV-NLuc (Fig. 1b).

To generate the *GJB1* P2 5’ UTR bicistronic vectors, pCMV-FLuc-*GJB1* P2 5’UTR-NLuc, the genomic region coding for the *GJB1* P2 5’ UTR (chrX:71,223,206–71,223,707 hg38) was amplified from a patient with the *GJB1* c.-103C > T mutation using GJB1P25UTR_HindIII_FGJB1P25UTR_XhoI_R (Table 2). The amplicon was cloned into the bicistronic pCMV-FLucNLuc vector in the intercistronic region between FLuc and NLuc using the HindIII/XhoI sites. Q5 SDM was used to correct the *GJB1* c.-103C > T mutation to wild type using GJB1_WTSDM_F and GJB1_WTSDM_R (Table 2) and the *GJB1* c.-102G > A polymorphism was introduced using GJB1_102GASDM_F and GJB1_102GASDM_R.

Vectors without the *GJB1* intron (Fig. 1d) were also generated for the wild-type *GJB1* P2 5’ UTR, *GJB1* c.-103C > T 5’ UTR and *GJB1* c.-102G > A using primers flanking the 356 bp intron, *GJB1*_Introndel_F and *GJB1*_Introndel_R (Table 2).

To generate a promoterless control (Fig. 1c (i) and d (i)) for the assessment of background luminescence (Fig. 1a), the CMV promoter of the pCMV-FLuc-*GJB1*P25’UTR-NLuc vectors (both with and without the intron) was deleted using primers which flank the CMV promoter region, CMVPromdel_F and CMVPromdel_R (Table 2).

### Generation of pGL4-based reporter luciferase vectors

The promoterless pGL4.10[luc2] luciferase reporter vector was used to generate a suite of reporter constructs to assess the expression of the *GJB1* P2 promoter and 5’ UTR. The *GJB1* P2 promoter and 5’ UTR (hg38-chrX:71,222,954–71,223,707) were amplified from control genomic DNA using GJB1P2Prom5UTR_HindIII_F and GJB1P2Prom5UTR_XhoI_R (Table 2). This amplicon was inserted between the HindIII and XhoI restriction sites in the pGL4.10[luc2] vector. The *GJB1* 3’ UTR (hg38-chrX:71,224,560–71,225,516) was amplified from control genomic DNA using GJB13UTR_F and GJB13UTR_R (Table 2). This amplicon was inserted between the XbaI and FseI sites in the pGL4.10[luc2] vector with the *GJB1* P2 promoter and 5’ UTR previously inserted. For the *GJB1* 5’ UTR to directly control the translation of FLuc, Q5 SDM was utilised to delete the region encompassing the HindIII recognition site to the base preceding the FLuc start codon using HindIII_ATGdel_F and HindIII_ATGdel_R (Table 2).

This vector was then used as a template for Q5 SDM to introduce the c.-103C > T mutation using GJB1_103CTSDM_F and GJB1_103CTSDM_R (Table 2), the c.-102G > A variant using GJB1_102GASDM_F and GJB1_102GASDM_R (Table 2), the c.-131A > G variant using GJB1_131AGSDM_F and GJB1_131AGSDM_R (Table 2) and the c.-108_-103del variant using GJB1_108103delSDM_F and GJB1_108103delSDM_R (Table 2).

### Cell culture

Rat Schwann cells (RT4) were cultured in a 96-well plate in Dulbecco’s Modified Eagle’s Medium (DMEM: Gibco) with 10% (v/v) foetal bovine serum (Gibco) at 37 °C with 5% CO_2_ until they reached 70–80% confluency. HeLa cells were cultured in a 96-well plate in DMEM (Gibco) with 10% (v/v) foetal bovine serum (Gibco) and 2 mM l-glutamine (Gibco) at 37 °C with 5% CO_2_ until they reached 80–90% confluency.

### Bicistronic assay transfection

Cells were transiently transfected with 100 ng per well of a bicistronic vector using Lipofectamine 3000 (Invitrogen) according to the manufacturer’s instructions. All bicistronic assays were repeated in triplicate, with each vector transfected into four wells for each assay. Each bicistronic assay was conducted with the negative IRES control (Fig. 1a) and the positive EMCV control (Fig. 1b). The *GJB1* bicistronic vectors containing the 356 bp *GJB1* intron (Fig. 1c) were tested in the RT4 cell line, and the *GJB1* bicistronic vectors containing the 356 bp *GJB1* intron (Fig. 1d) were tested in both the RT4 Schwann cell line and HeLa cell line.

### Bicistronic assay

A dual-luciferase assay was performed 48 h post-transfection using the NanoGlo Dual Luciferase Assay (Promega) according to the manufacturer’s instructions for 96-well plates using multichannel pipettes. FLuc and NLuc activity was measured using a luminometer (Perkin Elmer Enspire II) 15 min post the addition of their respective substrates. Luminescence was normalised against the appropriate promoterless vector and the ratio of NLuc:FLuc was calculated. Using these values, the relative response ratios (RRR) were calculated where the ratio of NLuc:FLuc for the positive EMCV IRES control was set to a value of 1 and the ratio of NLuc:FLuc for the negative IRES control was set to a value of 0 using the following equation:

$$RRR=\frac{experimental\ ratio- negative\ IRES\ ratio}{positive\ EMCV\ ratio- negative\ IRES\ ratio}$$

Statistical significance was assessed using a one-way ANOVA with a p-value < 0.05 suggesting statistical significance.

### RNA extraction and analysis

Cultured rat Schwann cells (RT4) were grown in a 6-well plate in Dulbecco’s Modified Eagle’s Medium (DMEM) with 10% (v/v) foetal bovine serum until they reached 70–80% confluency. These cells were then transiently transfected with 2000 ng of a bicistronic assay vector using Lipofectamine 3000 (Invitrogen) according to manufacturer’s instructions. The same experimental procedure was repeated using HeLa cells, which were cultured in a 96-well plate in Dulbecco’s Modified Eagle’s Medium (DMEM) with 10% (v/v) foetal bovine serum and 2 mM l-glutamine (Gibco) at 37 °C with 5% CO_2_ until they reached 80–90% confluency. RNA was extracted from the cell lines 48 h post-transfection using the RNEasy Mini Kit (Qiagen). RNA was then reverse transcribed using iScript cDNA Synthesis Kit (Bio-Rad). A PCR amplification was performed using primers which amplify the complete bicistronic mRNA, which was sequenced using the BigDye Terminator Cycle Sequencing protocols at Garvan Molecular Genetics (Garvan Institute of Medical Research, Australia).

### pGL4-based reporter luciferase assays

RT4 cells were cultured as described previously in 96-well TC-treated plates. Cells were transiently transfected with 100 ng per well of a pGL4-based reporter vector using Lipofectamine 3000 (Invitrogen) according to the manufacturer’s instructions. Ten nanograms of pRL-TK (Promega) was co-transfected and RLuc expression was used as a transfection and cell viability control. All bicistronic assays were repeated in triplicate, with each vector transfected into three wells for each assay. Each assay was conducted with the wild-type *GJB1*-FLuc vector as a positive control and an empty pGL4 vector as the negative control.

A dual-luciferase assay was performed 48 h post-transfection using the Dual-Glo® Dual Luciferase Assay (Promega) according to manufacturer’s instructions for 96-well plates using multichannel pipettes. FLuc and RLuc activity was measured using a luminometer (Perkin Elmer Enspire II) 15 min after the addition of their respective substrates. Luminescence was normalised against the background and the ratio of FLuc:RLuc was calculated. Using these values, the relative response ratios (RRR) were calculated where the ratio of FLuc:RLuc for the wild-type *GJB1*-pGL4 vector was set to a value of 1 and the ratio of FLuc:RLuc for the empty pGL4 vector was set to a value of 0 using the following equation:

$$RRR=\frac{experimental\ ratio- empty\ pGL4\ ratio}{wild\ type\ GJB1- pGL4\ ratio- empty\ pGL4\ ratio}$$

Statistical significance was assessed using a two-tailed t-test with a p-value < 0.05 suggesting statistical significance.

## Supplementary Information

Below is the link to the electronic supplementary material.

Supplementary file1 (DOCX 12653 KB)

## Data Availability

Not applicable.
