# Supplementary material for: Revisiting the pathogenic mechanism of the GJB1 5’ UTR c.-103C > T mutation causing CMTX1
Source: Neurogenetics. 2021 Jun 5;22(3):149–60. doi: 10.1007/s10048-021-00650-9 (PMC8241655; doi:10.1007/s10048-021-00650-9)
Supplement: Supplementary file 1 — Supplementary file1 (DOCX 12653 KB) [file 10048_2021_650_MOESM1_ESM.docx]

**Revisiting the Pathogenic Mechanism of the *GJB1* 5’ UTR c.-103C>T mutation causing CMTX1**

Bianca R. Grosz^1,2^*, John Svaren^2,3,4^, Gonzalo Perez-Siles^1,2^, Garth A. Nicholson^1,2,5^, Marina L. Kennerson^1,2,5​^

(1) Northcott Neuroscience Laboratory, ANZAC Research Institute, Concord, NSW, Australia.

(2) Sydney Medical School, University of Sydney, Camperdown, NSW, Australia.

(3) Waisman Center, University of Wisconsin-Madison, Madison, WI, USA.

(4) Department of Comparative Biosciences, University of Wisconsin-Madison, Madison, WI, USA.

(5) Molecular Medicine Laboratory, Concord Repatriation General Hospital, Concord, NSW, Australia

*email: [bgro4046@uni.sydney.edu.au](mailto:bgro4046@uni.sydney.edu.au)

ORCID:

Bianca R. Grosz: 0000-0002-6926-0551

John Svaren: 0000-0003-2963-7921

Gonzalo Perez-Siles: 0000-0002-1010-6445

Garth A. Nicholson: 0000-0001-9694-066X

Marina L. Kennerson: 0000-0003-3332-5074


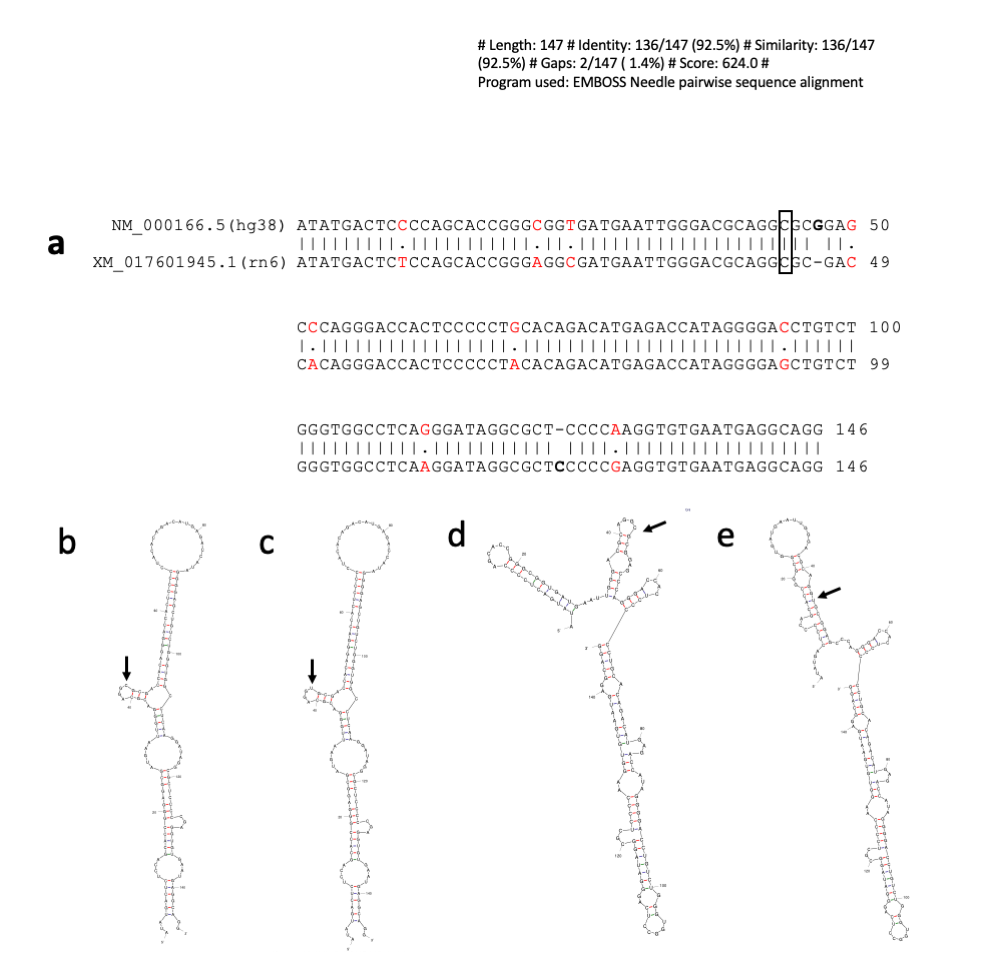


**Supplementary Figure 1:** (a) Sequence alignment of the human *GJB1* P2 5’ UTR (hg38) and the rat *GJB1* P2 5’ UTR (rn6) reveals 92.5% sequence similarity. Differing bases are indicated in red, insertions resulting in gaps are bolded. The site of the c.103>T mutation is boxed. (b-e) Wild type rat *GJB1* P2 5’ UTR (b) was predicted to generate the same RNA secondary structure as the rat *GJB1* c.-103C>T P2 5’ UTR (c). However, wild type human *GJB1* P2 5’ UTR (d) was predicted to result in a different RNA secondary structure when the c.-103C>T mutation (e) was introduced. Wild type human (d) and wild type rat (b) *GJB1* P2 5’ UTR also differ. The site of the c.-103C/T base is indicated with an arrow for each predicted RNA secondary structure.


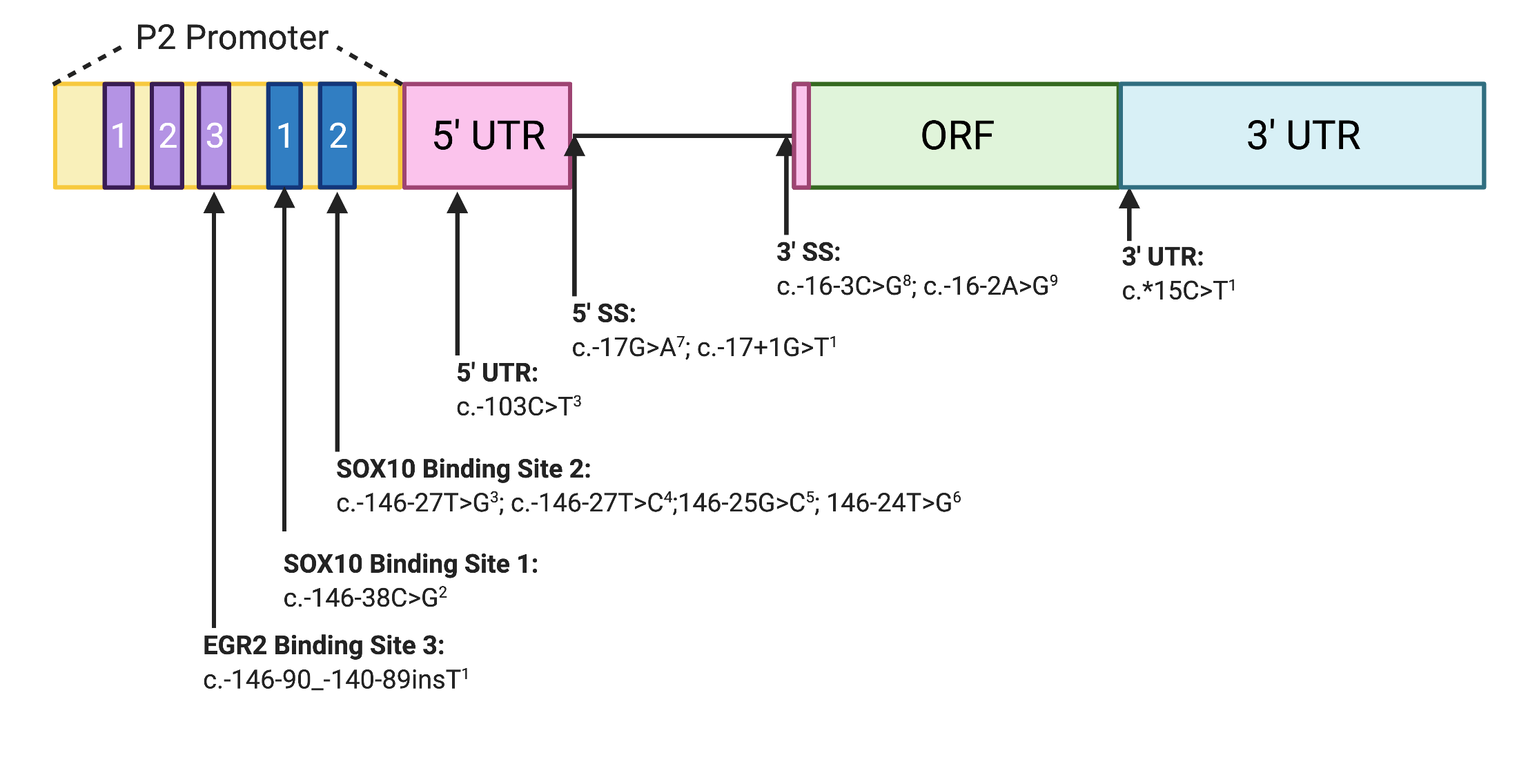


**Supplementary Figure 2: Structure of the P2 *GJB1* gene with reported non-coding mutations indicated.** The P2 promoter (yellow) consists of three EGR2 binding sites (purple) and two SOX10 binding sites (dark blue) to enable transcription of the P2 *GJB1* transcript. The P2 *GJB1* transcript is expressed in Schwann cells and oligodendrocytes and consists of the P2 5’ UTR (pink), the 356 bp intron (black line), the open reading frame (ORF) which encodes Cx32 (green), and the 3’ UTR (pale blue). Mutations are described using Human Genome Variation Society (HGVS) nomenclature. The associated reference is indicated in superscript. UTR: untranslated region; SS: splice site; ORF: open reading frame

1. Tomaselli PJ, Rossor AM, Horga A, et al (2017) Mutations in noncoding regions in *GJB1* are a major cause of X-linked CMT. Neurology 88:1445–1453.

2. Sivera R, Sevilla T, Vilchez JJ, Martinez-Rubio D, Chumillas MJ, Vazquez JF, Muelas N, Bataller L, Millan JM, Palau F, Espinos C. Charcot-Marie-Tooth disease: Genetic and clinical spectrum in a Spanish clinical series. Neurology 2013; 81, 1617-1625.

3. Ionasescu VV, Searby C, Ionasescu R, Neuhaus IM, Werner R. Mutations of the noncoding region of the connexin32 gene in X-linked dominant Charcot-Marie-Tooth neuropathy. Neurology 1996;47:541-544.

4. Beauvais K, Furby A, Latour P. Clinical, electrophysiological and molecular genetic studies in a family with X-linked dominant Charcot-Marie-Tooth neuropathy presenting a novel mutation in *GJB1* Promoter and a rare polymorphism in LITAF/SIMPLE. Neuromuscul Disord 2006;16:14-18.

5. Houlden H, Girard M, Cockerell C, et al. Connexin 32 promoter P2 mutations: a mechanism of peripheral nerve dysfunction. Ann Neurol 2004;56:730-734.

6. Luo S, Jin H, Chen J, Zhang L. A Novel Variant in Non-coding Region of *GJB1* Is Associated With X-Linked Charcot-Marie-Tooth Disease Type 1 and Transient CNS Symptoms. Front Neurol. 2019 Apr 24;10:413.

7. Murphy SM, Polke J, Manji H, et al. A novel mutation in the nerve-specific 5'UTR of the *GJB1* gene causes X-linked Charcot-Marie-Tooth disease. J Peripher Nerv Syst 2011;16:65-70.

8. Benedetti S, Previtali SC, Coviello S, et al. Analyzing histopathological features of rare Charcot-Marie-Tooth neuropathies to unravel their pathogenesis. Arch Neurol 2010;67:1498-1505.

9. Gonzaga-Jauregui C, Harel T, Gambin T, Kousi M, Griffin LB, Francescatto L, Ozes B, Karaca E, Jhangiani SN, Bainbridge MN, Lawson KS, Pehlivan D, Okamoto Y, Withers M, Mancias P, Slavotinek A, Reitnauer PJ, Goksungur MT, Shy M, Crawford TO, Koenig M, Willer J, Flores BN, Pediaditrakis I, Us O, Wiszniewski W, Parman Y, Antonellis A, Muzny DM; Baylor-Hopkins Center for Mendelian Genomics, Katsanis N, Battaloglu E, Boerwinkle E, Gibbs RA, Lupski JR. Exome Sequence Analysis Suggests that Genetic Burden Contributes to Phenotypic Variability and Complex Neuropathy. Cell Rep. 2015 Aug 18;12(7):1169-83.
